# VALIDITY OF COMMUNITY-BASED FRAILTY CHECK-UP BY SENIOR VOLUNTEERS FOR PREDICTING ADVERSE HEALTH OUTCOMES

**DOI:** 10.1093/geroni/igz038.2514

**Published:** 2019-11-08

**Authors:** Tomoki Tanaka, Kyo Takahashi, Masahiro Akishita, Katsuya Iijima

**Affiliations:** 1 Department of Geriatric medicine, The University of Tokyo, Hongo, Bunkyo-ku, Japan; 2 Institute of Gerontology, The University of Tokyo, Hongo, Bunkyo-ku, Japan; 3 Department of Geriatric Medicine, Graduate School of Medicine, the University of Tokyo, Tokyo, Japan; 4 Institute of Gerontology, The University of Tokyo, Tokyo, Japan

## Abstract

Aim: For achieving healthy aging for all, multi-faceted frailty is serious problem in super-aged society such as Japan. We developed community-based frailty check-up program performed by trained senior volunteers. In this study, we aimed to validate the ability of the results of check-up to predict needing long-term support or care insurance or death in community-dwelling older population. Methods: A total of 1,536 older adults (mean age, 73.0±6.1 years; 74% women; non-eligible for long-term support or care) participated in the check-ups held from April, 2015 to March, 2018 in Kashiwa City, Japan. At check-ups cite, 21 items including nutrition, oral and physical functions, and social conditions were assessed; Outcome was needing long-term support or care insurance, or death from the day of check-ups until October, 2018. Results: During follow-up {median 678 days (inter-quartile range, 199-1263)}, 116 (7.6%) were newly needing for long-term support (n=50) or care (n=49), or death (n=18). The number of positive responses among 21 items was associated with decreased risks of outcome {age-sex adjusted hazard ratio (95% confidence interval), 0.87 (0.81-0.92)}. Compared those with > 18 positive responses (third tertile), individuals with < 14 positive responses (first tertile) were highly increased risks of outcome {age-sex adjusted hazard ratio (95% confidence interval), 2.44 (1.22-4.49)}. Conclusions: Community-based frailty check-ups program could predict the needing long-term support or care insurance or death in community-dwelling older population. The appropriate intervention for individuals with bad results of the check-up might contribute to serving as early prevention of multi-faceted frailty.

